# Demographic Factors Associated With Perceptions About Water Safety and Tap Water Consumption Among Adults in Santa Clara County, California, 2011

**DOI:** 10.5888/pcd11.130437

**Published:** 2014-06-12

**Authors:** Brianna van Erp, Whitney L. Webber, Pamela Stoddard, Roshni Shah, Lori Martin, Bonnie Broderick, Marta Induni

**Affiliations:** Author Affiliations: Whitney L. Webber, Pamela Stoddard, Roshni Shah, Lori Martin, Bonnie Broderick, Santa Clara County Public Health Department, San Jose, California; Marta Induni, Survey Research Group, Public Health Institute, Sacramento, California.

## Abstract

The objective of this study was to examine differences in tap water consumption and perceptions of bottle versus tap water safety for Hispanics and non-Hispanic whites, as well as associations with other demographic characteristics. Data are from the Santa Clara County, California, Dietary Practices Survey (2011; N = 306). We used logistic regression to examine associations between demographic characteristics and 1) perceptions that bottled water is safer than tap and 2) primarily consuming tap water. Hispanics were less likely than non-Hispanic whites to primarily drink tap water (OR = 0.33; 95% CI, 0.11–0.99), although there was no significant difference in perceptions that bottled water is safer between these groups (OR = 0.50; 95% CI, 0.11–2.27). Hispanics may be an important population for interventions promoting tap water consumption.

## Objective

Although total water consumption in the US comes primarily from tap water, a significant percentage comes from bottled water (1). Choosing bottled water over tap water may be related to perceptions of safety, taste, and convenience (2–13). Understanding water consumption and perceptions of water safety can inform interventions like the Centers for Disease Control and Prevention’s (CDC) Communities Putting Prevention to Work (CPPW), which promotes tap water as a healthful, fluoridated, low-cost, and environmentally friendly alternative (14). Research has suggested that tap water consumption is lower among Hispanics, younger adults, and women (1,5,9,11). Hispanics may perceive tap water as unsafe given water quality issues in countries of origin among the foreign-born and issues stemming from older plumbing and contamination, especially among those living in lower-income housing or agricultural areas (3,15). In addition, populations with lower socioeconomic status may perceive tap water as less safe (3,4,6,8–11). Previous studies have relied on convenience samples (3–6,9–11,13) and qualitative data (3,4,9,10,12), have been descriptive (1,3,6,9–13,16,17), have focused on water intake only (1,16,18), have included only children (2,6,18,19), or did not include race/ethnicity (8,10–12,15). Our objective was to examine differences in tap water consumption and perceptions of bottled versus tap water safety for Hispanics and non-Hispanic whites, as well as associations with other demographic characteristics, by using a random and representative sample of adults.

## Methods

We used data from the Santa Clara County Dietary Practices Survey administered in 2011. The survey was funded by CPPW to inform Santa Clara County interventions to promote tap water consumption. The survey used the California Dietary Practices Survey, a statewide, cross-sectional, random-digit-dial telephone survey of noninstitutionalized adults conducted since 1989, adding a module on drinking water behaviors for Santa Clara County respondents (N = 368). The response rate was 22% and the cooperation rate was 47%. The survey was conducted by the Survey Research Group (Sacramento, California). Specific questions in this analysis were from New York City’s Public Opinion Survey on SSBs, Water, and Other Policy Initiatives and underwent cognitive testing before administration (20).

Outcome variables were 1) thinks bottled water is safer, and 2) primarily drinks tap water. Respondents were asked “Which do you think is safer, bottled water or Santa Clara County tap water or are they about the same?” Responses were categorized as 1) thinks bottled water is safer and 2) does not think bottled water is safer. Respondents also reported the type of water they consumed most often on a typical day. Responses were categorized as 1) primarily drinks tap water (unfiltered tap or filtered tap) or 2) primarily drinks bottled plain water or seltzer (soda) water.

Independent variables included sex, age, education, race/ethnicity, nativity, and income. Race/ethnicity was categorized as non-Hispanic white (hereafter, white), Hispanic, and other (African American, Alaska Native, American Indian, Asian, Pacific Islander, other). Categories for age, education, and income were based on the distribution of responses to ensure sufficient sample size for analysis.

The analytic sample included 306 adults. Respondents with missing data on any variable in the analysis (n = 62) were excluded. Those with missing data tended to be female, older, more educated, US-born, lower-income, and white; did not think bottled water was safer; and primarily drank tap water. Differences were not statistically significant. See Table 1 for a comparison of characteristics of the analytic sample to the Santa Clara County population. We used logistic regression (weighted) to examine determinants of perceptions of water safety and tap water consumption. Model 1 for tap water consumption included only sociodemographic characteristics; model 2 adjusted for safety perceptions.

**Table 1:** Sample Characteristics, Santa Clara County (SCC) Dietary Practices Survey, 2011 (N = 306)

Characteristic	n	%^a^	SCC Adult Demographic, %^b^
**Sex**			
Male	130	43	50
Female	176	58	50
**Age, y**			
18–44	101	33	52
45–64	129	42	33
≥65	76	25	15
**Education**			
High school graduate or less	62	20	31
Some college, associate’s degree, or more	244	80	69
**Nativity**			
US-born	211	69	54
Foreign-born	95	31	46
**Household income, $**			
<50,000	91	30	31
≥50,000	215	70	69
**Race**			
Non-Hispanic white	199	65	38
Hispanic	48	16	24
Other	59	19	38
**Water safety**			
Thinks bottled water is safer	81	27	—
Does not think bottled water is safer	225	74	—
**Water consumption**			
Primarily drinks tap water	218	71	—
Does not primarily drink tap water	88	29	—

a Some percentages may not sum to 100% because of rounding.

b Santa Clara County demographic estimates are from the US Census Bureau, American Community Survey 2011, 1-Year Estimates (22).

This study was certified as exempt from review by the Santa Clara County Health Services Institutional Review Board.

## Results

Respondents were more likely to be female, aged 45 to 64 years, and white (Table 1). Most respondents had some college education or more, an annual household income of $50,000 or more, and were US-born. Twenty-nine percent of adults thought bottled water was safer than tap, 68% primarily drank tap water, and 99% overall drank any water the previous day (weighted, not shown).

Adults with some college or more were less likely than those with a high school degree or less to think bottled water is safer (odds ratio [OR], 0.32; 95% confidence interval [CI], 0.11–0.91) (Table 2). The foreign-born were marginally more likely to think bottled water is safer (OR, 2.06; 95% CI, 0.89–4.77). Hispanics (OR, 0.36; 95% CI, 0.13–1.02) and foreign-born adults (OR, 0.43; 95% CI, 0.18–1.04) were marginally less likely to primarily drink tap water than whites or US-born adults, adjusting for other sociodemographic characteristics; after adjustment for perceptions of safety, Hispanics were significantly less likely than whites to primarily consume tap water (OR, 0.33; 95% CI, 0.11–0.99); results for the foreign-born remained marginally significant (OR, 0.48; 95% CI, 0.20–1.15). Adjusting for other characteristics, adults who think bottled water is safer were less likely to primarily drink tap water (OR, 0.28; 95% CI, 0.12–0.62).

**Table 2:** Odds of Perceptions of Water Safety and Tap Water Consumption, Santa Clara County Dietary Practices Survey, 2011 (N = 306)

Characteristic	Thinks Bottled Water is Safer		Primarily Drinks Tap Water			
	OR (95% CI)	*P* Value	OR (95% CI)	*P* Value	OR (95% CI)	*P* Value
**Sex**						
Male	1 [Reference]	—	1 [Reference]	—	1 [Reference]	—
Female	0.79 (0.39–1.58)	.497	0.75 (0.38–1.49)	0.413	0.76 (0.37–1.57)	.46
**Age, y**						
18–44	1 [Reference]	—	1 [Reference]	—	1 [Reference]	—
45–64	1.44 (0.67–3.09)	.354	0.53 (0.23–1.22)	0.134	0.58 (0.25–1.37)	.21
≥65	0.85 (0.29–2.46)	.758	0.74 (0.29–1.92)	0.533	0.91 (0.36–2.30)	.84
**Education**						
High school graduate or less	1 [Reference]	—	1 [Reference]	—	1 [Reference]	—
Some college, associate’s degree, or more	0.32 (0.11–0.91	.033	2.26 (0.65–7.92)	0.201	2.10 (0.57–7.81)	.27
**Nativity**						
US-born	1 [Reference]	—	1 [Reference]	—	1 [Reference]	—
Foreign-born	2.06 (0.89–4.77)	.092	0.43 (0.18–1.04	0.060	0.48 (0.20–1.15)	.10
**Income, $**						
<50,000	1 [Reference]	—	1 [Reference]	—	1 [Reference]	–
≥50,000 or higher	0.75 (0.27–2.14)	.595	0.69 (0.27–1.79)	0.443	0.66 (0.25–1.78)	.41
**Race**						
Non-Hispanic white	1 [Reference]	—	1 [Reference]	—	1 [Reference]	—
Hispanic	0.50 (0.11–2.27)	.366	0.36 (0.13–1.02	0.055	0.33 (0.11–0.99)	.048
Other	3.49 (1.46–8.29)	.005	1.29 (0.47–3.53	0.617	2.31 (0.76–7.07)	.14
**Water safety**						
Does not think bottled water is safer	—	—	—	—	1 [Reference]	—
Thinks bottled water is safer	—	—	—	—	0.28 (0.12–0.62)	.002

Abbreviation: —, not applicable.

## Discussion

Most adults (68%) in Santa Clara County, a diverse, urban county in Northern California, primarily consume tap water. Adjusting for other characteristics, Hispanic adults are less likely to primarily consume tap water than whites. This finding is consistent with a descriptive study based on a national sample, which found that Mexican Americans consumed more bottled than tap water while the opposite pattern was true for whites (1). Another study based on a convenience sample found that Hispanic parents were less likely than non-Hispanic parents to drink tap water (5). Several studies of children or of parent provision of water to children had similar findings (1,4,18).

Although most adults in Santa Clara County primarily consume tap water, nearly 3 in 10 believe that bottled water is safer. Unlike previous studies, we found no difference between Hispanics and whites concerning perceptions of safety of tap versus bottled water, which may be due to differences in question phrasing (2,4,5). Perceptions of safety did not explain differences in water consumption for Hispanics versus whites, and, in fact, differences were only apparent after controlling for safety perceptions; this difference in consumption may be due to factors such as taste, access, or custom. It is unlikely to be due to cost, since tap water is much less expensive.

Consistent with previous studies, less educated adults were more likely to think bottled water is safer, and perceptions that bottled water was safer had an independent, negative effect on primarily consuming tap water (3,4,6,8–11).

This study was subject to limitations. The survey included only households with landline telephones; Hispanics are more likely to live in wireless-only households (21). The study was situated in a single county, which may limit generalizability to other settings.

Several interventions, such as Santa Clara County’s Rethink Your Drink and Water to Go campaigns, encourage water consumption (http://www.cdph.ca.gov/programs/wicworks/Pages/WICRethinkYourDrink.aspx, http://www.sccgov.org/sites/sccphd/en-us/Newsandevents/Documents/Water/Water%20To%20Go%202013/FAQ_WaterToGo_Oct2013.pdf). Findings suggest that Hispanics are an important target population for such initiatives, especially for efforts promoting tap water consumption.
